# Treatment with the anti-IL-6 receptor antibody attenuates muscular dystrophy via promoting skeletal muscle regeneration in dystrophin-/utrophin-deficient mice

**DOI:** 10.1186/s13395-017-0140-z

**Published:** 2017-10-27

**Authors:** Eiji Wada, Jun Tanihata, Akira Iwamura, Shin’ichi Takeda, Yukiko K. Hayashi, Ryoichi Matsuda

**Affiliations:** 1 Department of Pathophysiology, Tokyo Medical University, 6-1-1 Shinjuku, Shinjuku, Tokyo, Japan; 2 Department of Life Sciences, Graduate School of Arts and Sciences, The University of Tokyo, 3-8-1 Komaba, Meguro, Tokyo, Japan; 3 Department of Molecular Therapy, National Institute of Neuroscience, National Center of Neurology and Psychiatry, 4-1-1 Ogawa-Higashi, Kodaira, Tokyo, Japan; 4 Department of Cell Physiology, The Jikei University School of Medicine, 3-25-8, Nishi-Shimbashi, Minato-ku, Tokyo, Japan; 5 Public Health, Department of Social Medicine, Osaka University Graduate School of Medicine, 2-2 Yamadaoka, Suita, Osaka, Japan

**Keywords:** Interleukin-6, Duchenne muscular dystrophy, STAT3, Muscle regeneration, Fibrosis

## Abstract

**Background:**

Chronic increases in the levels of the inflammatory cytokine interleukin-6 (IL-6) in serum and skeletal muscle are thought to contribute to the progression of muscular dystrophy. Dystrophin/utrophin double-knockout (dKO) mice develop a more severe and progressive muscular dystrophy than the mdx mice, the most common murine model of Duchenne muscular dystrophy (DMD). In particular, dKO mice have smaller body sizes and muscle diameters, and develop progressive kyphosis and fibrosis in skeletal and cardiac muscles. As mdx mice and DMD patients, we found that IL-6 levels in the skeletal muscle were significantly increased in dKO mice. Thus, in this study, we aimed to analyze the effects of IL-6 receptor (IL-6R) blockade on the muscle pathology of dKO mice.

**Methods:**

Male dKO mice were administered an initial injection (200 mg/kg intraperitoneally (i.p.)) of either the anti-IL-6R antibody MR16-1 or an isotype-matched control rat IgG at the age of 14 days, and were then given weekly injections (25 mg/kg i.p.) until 90 days of age.

**Results:**

Treatment of dKO mice with the MR16-1 antibody successfully inhibited the IL-6 pathway in the skeletal muscle and resulted in a significant reduction in the expression levels of phosphorylated signal transducer and activator of transcription 3 in the skeletal muscle. Pathologically, a significant increase in the area of embryonic myosin heavy chain-positive myofibers and muscle diameter, and reduced fibrosis in the quadriceps muscle were observed. These results demonstrated the therapeutic effects of IL-6R blockade on promoting muscle regeneration. Consistently, serum creatine kinase levels were decreased. Despite these improvements observed in the limb muscles, degeneration of the diaphragm and cardiac muscles was not ameliorated by the treatment of mice with the MR16-1 antibody.

**Conclusion:**

As no adverse effects of treatment with the MR16-1 antibody were observed, our results indicate that the anti-IL-6R antibody is a potential therapy for muscular dystrophy particularly for promoting skeletal muscle regeneration.

**Electronic supplementary material:**

The online version of this article (10.1186/s13395-017-0140-z) contains supplementary material, which is available to authorized users.

## Background

Duchenne muscular dystrophy (DMD) is the most common form of muscular dystrophy worldwide [1]. DMD is caused by mutations in the *DMD* gene on chromosome Xp21 encoding a subsarcolemmal large protein named dystrophin. A lack of dystrophin in skeletal and cardiac muscles results in progressive muscle degeneration, cardiac or respiratory complications, and early death [2]. At present, there is no curative therapy for DMD patients. In DMD, loss of dystrophin leads to muscle fiber damage and subsequent regeneration in which satellite cells (muscle stem cells) play an indispensable role. Prolonged muscle degeneration and regeneration impedes satellite cell activation and increases fat and/or fibrotic tissue replacement. Inflammatory cells are known to contribute to the progression of the dystrophic phenotypes in chronic disease, with fatty and fibrotic tissue replacement [3]. Therefore, the skeletal muscle in DMD patients is eventually replaced with non-functional tissues [4, 5], and hence preventing the accumulation of connective and adipose tissues is an important factor for delaying disease progression.

Several inflammatory factors are increased in the DMD skeletal muscle, including interleukin-6 (IL-6), TNF-alpha, and NF-kappaB [6]. IL-6 is mainly produced by T cells and macrophages to stimulate the immune response (pro-inflammatory), and an increase in IL-6 levels is an important contributor of the pathogenesis of inflammatory diseases [7]. IL-6 plays multiple biological roles in different signaling pathways through the IL-6 receptor (IL-6R) and activates downstream intracellular signaling cascades including the Janus kinase/signal transducer and activator of transcription (JAK/STAT) pathway.

IL-6 is secreted from different types of cells including muscle cells [8]. In the healthy skeletal muscle, IL-6 plays anti-inflammatory roles, and its levels were found to increase in response to exercise without any sign of muscle damage [9]. In the skeletal muscle, acute treatment with high-dose IL-6 leads to muscle breakdown in rats, and long-term IL-6 infusion results in muscle atrophy [10, 11]. The pro-inflammatory role of IL-6 in DMD was previously reported by studying both human and dystrophin-deficient mdx mice. Serum levels of IL-6 in DMD patients and mdx mice are significantly increased compared with healthy controls [12], and the levels gradually increase with age and disease progression [6]. Pelosi et al. crossed mdx mice and IL-6 transgenic mice to generate mdx/IL-6 transgenic mice, which showed a more severe dystrophic phenotype than mdx mice [13]. These previous studies demonstrated that IL-6-mediated immunological responses may promote additional muscle fiber damage under conditions of dystrophin deficiency.

A recombinant humanized monoclonal IL-6R antagonist (tocilizumab) has been approved as an anti-inflammatory drug for inflammatory diseases such as rheumatoid arthritis, Castleman’s disease, and systemic juvenile idiopathic arthritis. Tocilizumab blocks IL-6-mediated signaling via inhibiting the binding of IL-6 to both soluble and transmembrane IL-6Rs. Thus, treatment of anti-IL-6R blockade has the potential to inhibit the progression of DMD. However, previous reports showed controversial results on the effectiveness of a rat anti-mouse IL-6R antibody (MR16-1) using mdx mice. Kostek et al. showed that there were no therapeutic effects on muscle pathology [14] whereas another study demonstrated an improvement in muscle pathology and function [15]. These controversial results of MR16-1 treatment on mdx mice hinder the use of IL-6R antagonists for the treatment of DMD patients, and it is therefore necessary to perform further studies to clarify the effects.

The mdx mouse model, which is the most common murine DMD model, is widely used for analyzing the pathology of DMD; however, mdx mice have a mild muscle pathology with less fat and fibrosis accumulation [16]. This mild DMD phenotype is partly due to an upregulation of utrophin, a homolog of dystrophin that partially compensates for the function of dystrophin [17, 18]. In addition, mdx mice have a high capacity of muscle fiber regeneration, and even muscle stem cells (satellite cells) from aged mdx mice retain their regenerating capacity [19]. Compared to mdx mice, dystrophin and utrophin double-knockout (dKO) mice display a severer dystrophic phenotype including marked muscle fiber degeneration followed by the accumulation of connective tissue, smaller muscle fiber diameter, cardiac dysfunction, and abnormal spinal curvature [20, 21]. Therefore, dKO mice are much more suitable for examining the therapeutic efficacy of potential drugs for dystrophin deficiency. In this study, we investigated the effects of MR16-1 antibody administration on the dystrophic phenotypes of dKO mice, and confirmed that IL-6R blockade has therapeutic effects on the dystrophic skeletal muscle.

## Methods

### Animal care and drug treatment

The dKO (mdx of C57BL/6 background/utrophin−/−) mice used in this study were established from mdx/utrophin+/− breeder pairs, and dKO mice and C57BL/6J (wild-type) mice were maintained in a specific pathogen free facility. All experimental procedures were approved by the Experimental Animal Care and Use Committees of the National Institute of Neuroscience, National Center of Neurology and Psychiatry, Tokyo Medical University, and the University of Tokyo. Male mice were randomly assigned to either the anti-IL-6R antibody (MR16-1, kindly provided by Chugai Pharmaceutical Co., Ltd., Tokyo, Japan) or isotype-matched rat IgG control (Southern Biotechnology Association Inc., Birmingham, AL) injection groups. Drug administration was started at the age of 14 days of a single dose of 200 mg/kg i.p. to induce tolerance, and mice were given weekly injections of 25 mg/kg of MR16-1 or a rat IgG. Body weight was measured when mice were sacrificed at 90 days of age, and serum and muscle samples were collected for further analyses.

### Kyphotic index (KI)

After muscle samples and internal organs were removed, corpses of dKO mice were fixed using 10% formalin/PBS. To quantify spinal curvature, whole-body radiographs of dKO mice were captured using a Latheta LCT-200 X-ray micro CT scanner (Hitachi Ltd., Tokyo, Japan). The KI of each mouse was measured using a protocol established by Laws and Hoey [22]. Namely, the ratio of length 1 (L1) to length 2 (L2) corresponds to the KI, in which L1 is the direct distance between the seventh cervical vertebra (C7) to the postal edge of the sixth lumbar vertebra (L6), and L2 is the distance between the point of the distal border of the vertebra curvature to the perpendicular point of L1. Therefore, a smaller KI value indicates the progression of kyphosis.

### Serum creatine kinase (CK) levels

Blood samples were collected via the caudal vena cava, and serum samples were separated by incubation at room temperature for 2 h, followed by centrifugation at 3500 rpm for 15 min. Serum CK levels were measured using a biochemistry automatic analyzer (model 7180; Hitachi High-Tech, Tokyo, Japan).

### Serum and muscle IL-6 measurement

Serum samples were prepared as above. Quadriceps muscle samples were snap-frozen in cooled liquid nitrogen and homogenized with RIPA buffer (WAKO, Osaka, Japan) containing a protease inhibitor cocktail (Roche Diagnosis, Basel, Switzerland). Samples were centrifuged at 15,000 rpm for 10 min, and protein concentrations in the supernatants were analyzed using a Bradford protein assay kit (Bio-Rad, Hercules, CA). Serum and muscle IL-6 levels were measured using an ELISA kit according to the manufacturer’s instructions (R&D Systems, Minneapolis, MN).

### Quantitative RT-PCR

Muscle samples (quadriceps and cardiac) from rat IgG- and MR16-1-treated dKO mice and wild-type mice, and muscle samples (quadriceps, diaphragm, and cardiac) from non-treated dKO mice were homogenized using Trizol reagent (Life Technologies, Gaithersburg, MD). RNA was isolated with the PureLink RNA mini kit (Life Technologies) according to the manufacturer’s instructions. Before real-time PCR, total RNA (1 μg) was reverse-transcribed using superscript VILO cDNA synthesis kit (Invitrogen, Carlsbad, CA). Then, cDNA (25 ng) was amplified using SYBR Green PCR Master Mix (Applied Biosystems, Foster City, CA). The primer sequences used for gene expression analyses are listed in Additional file 1: Table S1 and all data were normalized using the internal control gene *Tbp* (encoding TATA box-binding protein) or *Lbr* (encoding lamin B receptor). Data were expressed as the fold increase versus the values of wild-type mice.

### Western blot analysis

Quadriceps and cardiac muscles were lysed in RIPA buffer containing protease inhibitors and phosphatase inhibitors (Roche). Samples were centrifuged at 15,000 rpm for 20 min, and supernatants were mixed with sample buffer solution (WAKO). Equal volumes of protein (60 μg) were separated on 5–20% gradient SDS-PAGE gels (WAKO) and transferred onto PVDF membranes using a semi-dry transfer apparatus (Bio-Rad). After blocking with 2% casein in TBS with 0.05% Tween-20, primary antibodies were applied to the membranes overnight at 4 °C. After washing, the membranes were incubated with horseradish peroxidase-conjugated secondary antibody. All bands were visualized using an ECL substrate solution with ChemiDoc imager (Bio-Rad). The band intensities of the target proteins were analyzed using the NIH ImageJ software and were normalized by the band intensity of GAPDH. The primary antibodies used in this study were as follows: anti-phospho-STAT3 (Tyr705, Cell Signaling Technology, Danvers, MA), anti-STAT3 (Cell Signaling Technology), anti-periostin (Novus Biologicals, Littleton, CO), anti-PDGFRα (R&D Systems), and anti-GAPDH (Sigma, St. Louis, MO).

### Histology and immunohistochemistry

Transverse 7-μm-thick cryosections of quadriceps, heart, and diaphragm muscles were collected and stained with hematoxylin and eosin (H&E) and Masson’s trichrome stains (Sigma). For immunohistochemistry, cryosections were fixed with 4% paraformaldehyde/PBS or cold acetone. After blocking with 0.2% BSA/PBS, samples were incubated with primary antibodies at 37 °C for 1 h. Alexa Fluor 488 or 568 secondary antibody (1:1000; Thermo Fisher Scientific, Waltham, MO) with DAPI solution was used for detection. Primary antibodies used were as follows: anti-F4/80 (Bio-Rad), anti-embryonic myosin heavy chain (eMyHC, F1.652, Developmental Studies Hybridoma Bank, Iowa City, IA), anti-Pax7 (Developmental Studies Hybridoma Bank), anti-laminin α2 (Enzo Life Sciences, Farmingdale, NY), anti-Ki67 (Thermo Fisher Scientific), anti-PDGFRα (R&D Systems), and anti-periostin (Novus Biologicals). Anti-mouse IgG (H + L) secondary antibody (Alexa Fluor 488) was used to detect necrotic muscle fibers.

### Muscle fiber diameter

Transverse cross-sections of the quadriceps muscle were stained with an anti-laminin α2 antibody and captured using an IN Cell Analyzer 2200 imaging system (GE Healthcare, Pittsburgh, PA). The minor axis of the fiber diameter was automatically calculated by the IN Cell Developer Toolbox software (GE Healthcare). A muscle fiber is first fit to a bounding ellipse. The shorter axis is automatically recognized as the minor axis and the longer is the major axis (Additional file 1: Figure S1). The minor axis was used to analyze a distribution of fiber diameter histograms of the quadriceps muscle from rat IgG, and MR16-1-treated dKO mice were compared.

### Statistical analysis

Results were expressed as means ± SD, and differences were determined by the Welch’s *t* test. For multiple comparisons, a one-way factorial ANOVA was performed with the SPSS Statistics ver. 22 software (SPSS, Chicago, IL). Statistical tests were two-sided, and *P* values ≤ 0.05 were considered to indicate a statistically significant difference between two groups. (*) represents *P* < 0.05, (**) represents *P* < 0.01, and (***) represents *P* < 0.001.

## Results

### Decreased serum CK levels and necrotic area by MR16-1

**Fig. 1:**
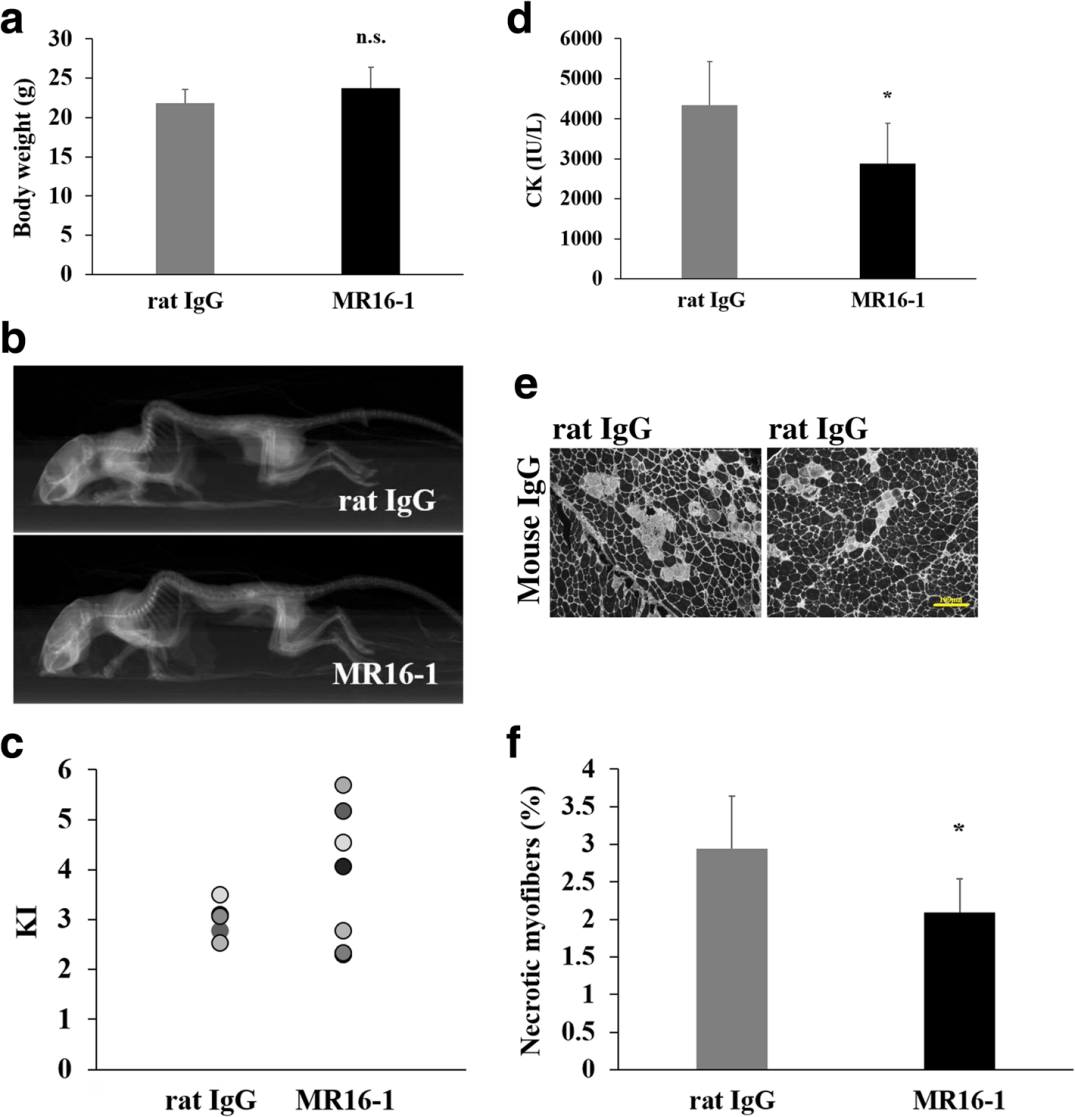
Body weight, KI, serum CK levels, and necrotic area in the quadriceps muscle. **a** Average body weight of mice at 90 days of age (*n* = 8 per group). **b** Radiographs of dKO mice treated with rat IgG or MR16-1. KI was measured using the radiographs. **c** KI of rat IgG dKO mice (*n* = 5) and MR16-1-treated dKO mice (*n* = 8). No significant difference was observed in the average KI between the two groups. **d** Serum CK levels of the mice at 90 days of age (*n* = 7–8). **e** A representative image of anti-mouse IgG-positive necrotic area in the quadriceps muscle. **f** The percentage of necrotic fibers/total fibers (× 200 magnification). Necrosis was significantly reduced in dKO mice by MR16-1 treatment. *P < 0.05

All rat IgG and MR16-1-treated dKO mice were alive until 90 days of age. Body weights were not significantly different between the two groups (21.9 ± 1.7 g in rat IgG vs. 23.7 ± 2.7 g in MR16-1-treated, *P* = 0.91, Fig. 1a). Body weights of both rat IgG and MR16-1-treated dKO mice were similar to that of non-treated dKO mice (data not shown). KI values were also not significantly different between the groups (3.0 ± 0.4 in rat IgG vs. 3.7 ± 1.3 in MR16-1-treated, *P* = 0.24, Fig. 1b, c); however, four mice from the MR16-1-treated group had relatively improved spinal curvature (KIs of 4.1, 4.5, 5.7, and 5.1). Serum CK levels of dKO mice were significantly reduced by MR16-1 treatment (4339.7 ± 1084.5 IU in rat IgG vs. 2883.8 ± 1005.5 IU in MR16-1-treated, *P* = 0.02, Fig. 1d). Decrease in serum CK levels was corroborated with a significant decrease in the percentage of necrotic area in the quadriceps muscle of MR16-1-treated dKO mice (*P* < 0.05, Fig. 1e, f).

### Successfully inhibited IL-6 signaling pathway in the dKO skeletal muscle by MR16-1

**Fig. 2:**
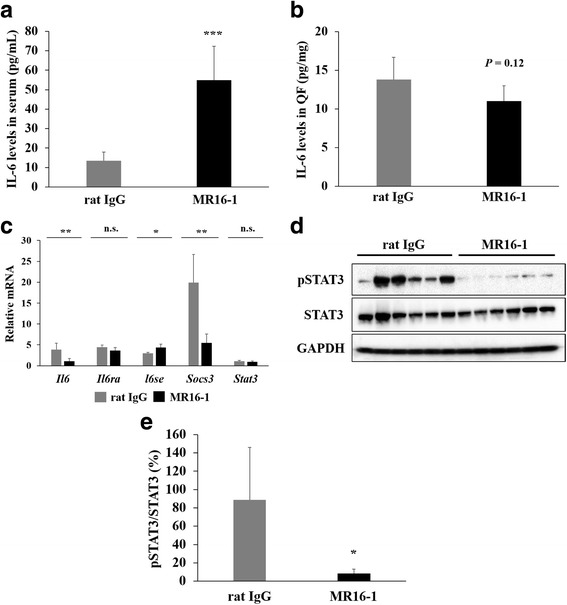
IL-6 levels in serum and the quadriceps muscle. **a** Serum IL-6 levels of mice at 90 days of age. Serum IL-6 levels of MR16-1-treated dKO mice were increased by about fourfold compared with the rat IgG group. **b** IL-6 levels of the quadriceps muscle (QF) of dKO mice treated with rat IgG or MR16-1. IL-6 levels were relatively lower in MR16-1-treated dKO mice (*P* = 0.12). **c** Quantitative RT-PCR analysis of the levels of *Il-6*, *Il-6ra*, *Il-6se*, *Socs3*, and *Stat3* were measured. All samples were normalized to the expression levels of *Lbr*. Values of rat IgG and MR16-1 groups were expressed as fold increase of wild-type mice (*n* = 3–4 per group). **d** A representative image of Western blot analysis of phosphorylated STAT3 (pSTAT3), total STAT3, and GAPDH. **e** The expression of pSTAT3 normalized by total STAT3 levels in dKO mice treated with rat IgG or MR16-1 (*n* = 6 per group). Activated STAT3 levels were significantly decreased in dKO mice by MR16-1 treatment. *P < 0.05, **P < 0.01, ***P < 0.001

Serum IL-6 levels were significantly increased in dKO mice treated with MR16-1 (13.5 ± 4.6 pg/mL in rat IgG vs. 55.0 ± 17.3 pg/mL in MR16-1-treated, *P* < 0.001, Fig. 2a) whereas IL-6 levels in the skeletal muscle were relatively decreased (*P* = 0.12, Fig. 2b). Muscle *Il6* gene expression was significantly reduced (*P* < 0.01, Fig. 2c), and the levels were similar with that of wild-type mice (the fold increase versus wild-type, 1.07 ± 0.63 in MR16-1-treated whereas 3.83 ± 1.54 in rat IgG). IL-6R blockade also effectively suppressed *Socs3* mRNA expression (*P* < 0.01, Fig. 2c). Gene expression levels of *Il6ra* were not affected but the levels of *Il6se* were significantly increased (*P* < 0.05, Fig. 2c) by MR16-1 treatment. *Stat3* mRNA and total STAT3 protein levels were not significantly different (Fig. 2c, d), whereas protein levels of phosphorylated STAT3 were significantly decreased (*P* < 0.05, Fig. 2e) by MR16-1 treatment.

### Improvement of muscle fiber diameters by MR16-1

**Fig. 3:**
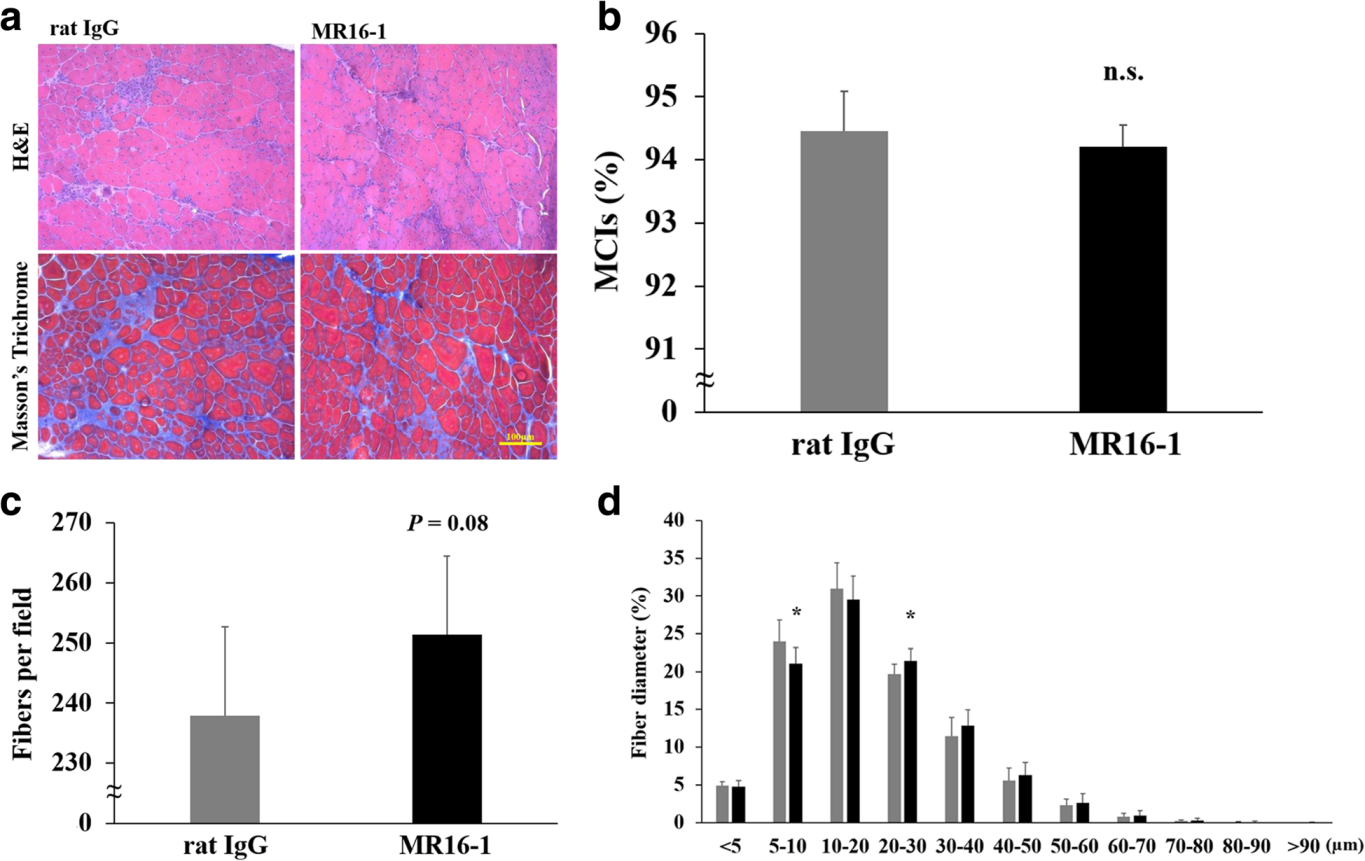
Histological analysis of the dystrophic phenotypes of the quadriceps muscle. **a** H&E and Masson’s trichrome staining of the quadriceps muscle from dKO mice. **b** The percentage of MCIs were counted. **c** Muscle fibers per field (× 200 magnification) were counted. MR16-1-treated dKO mice increased muscle fibers (*P* = 0.08). **d** Histogram of fiber diameters of the rectus femoris muscle (the largest part of the quadriceps). Data are expressed as a percentage of total fiber (*n* = 8 per group). *P < 0.05

Myofibers containing internal nuclei (MCIs) are generally recognized as muscle fibers that have regenerated after muscle damage. Approximately 1300–1600 myofibers were counted per section, and the quadriceps muscle from dKO mice treated with rat IgG contained 94.5% ± 0.6% MCIs whereas in MR16-1-treated dKO mice contained 94.2% ± 0.3% (Fig. 3a, b). There was a certain trend toward significance in the number of muscle fibers/field by MR16-1 treatment (*P* = 0.08, Fig. 3c). A histogram of fiber diameter was plotted by measuring the minor axis of fibers from quadriceps muscle. MR16-1 treatment improved the fiber diameter of dKO mice as the number of smaller fibers (5–10 μm) were significantly reduced (*P* < 0.05) and larger fibers (20–30 μm) were significantly increased (*P* < 0.05, Fig. 3d).

### Improvement of satellite cell function and muscle regeneration by MR16-1

**Fig. 4:**
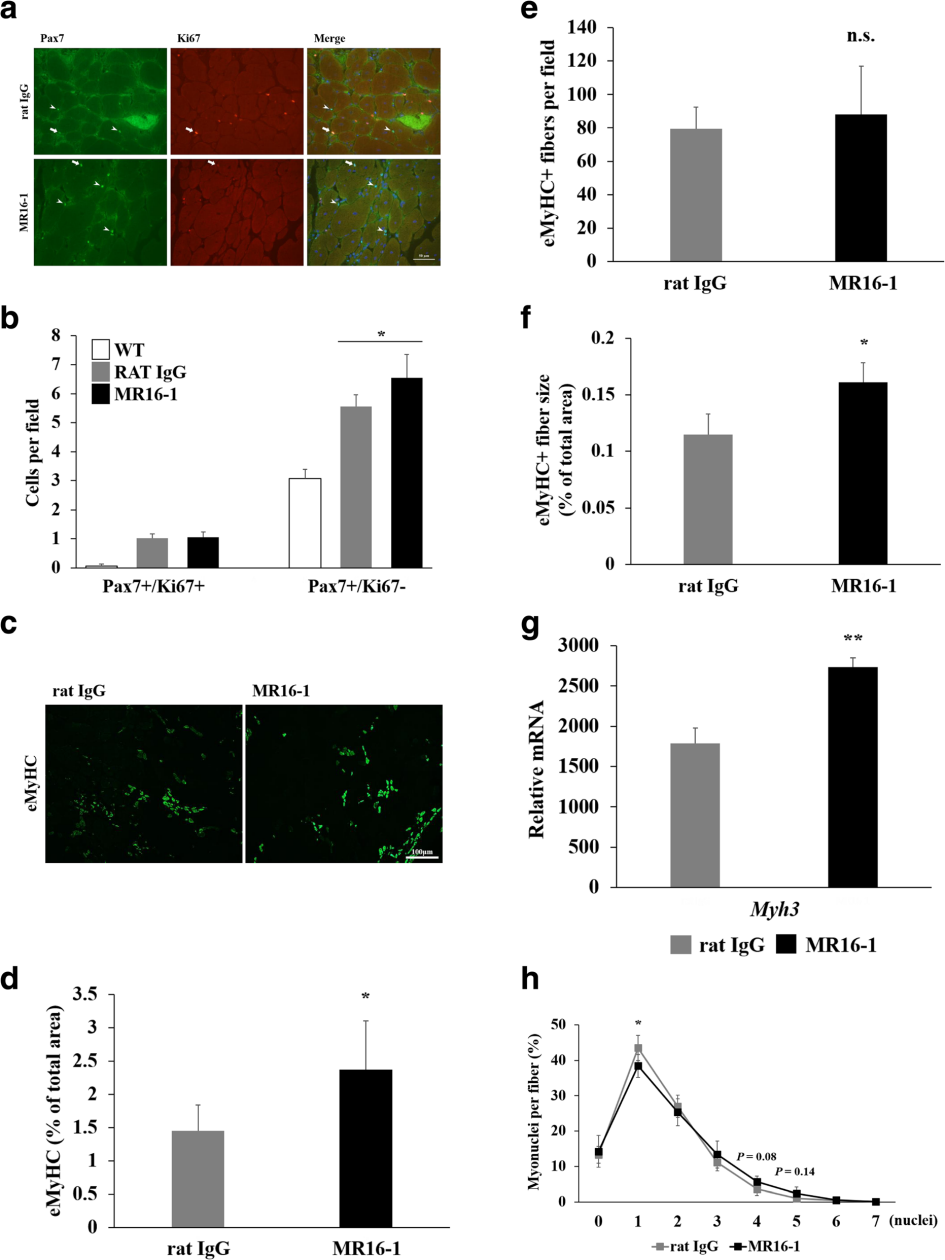
Satellite cell content and muscle regeneration. **a** Pax7 and Ki67 double staining of the quadriceps muscle sections. *Arrowheads* represent Pax7-positive and Ki67-negative quiescent satellite cells, and *arrows* represent double-positive (Pax7+/Ki67+) proliferated satellite cells. **b** The numbers of Pax7+/Ki67+ cells/field and Pax7+/Ki67− cells/field (×400 magnification) in wild-type and dKO mice treated with rat IgG or MR16-1 were compared. The greater number of both activated and quiescent satellite cells was found in dKO mice compared with wild-type mice. While the number of Pax7+/Ki67+ satellite cells was not different, Pax7+/Ki67− satellite cells were significantly increased in MR16-1-treated dKO mice (*P* < 0.05). **c** A representative image of eMyHC-positive regenerating muscle fibers in dKO mice treated with rat IgG or MR16-1. **d** The percentage of eMyHC-positive area per total area. **e** The number of eMyHC-positive regenerating muscle fibers per field (× 200 magnification). **f** The percentage of average eMyHC-positive muscle fiber size. **g** Gene expression levels of *myh3*, normalized by *Lbr*, was expressed as fold increase of wild-type mice (*n* = 3–4 per group). **h** The percentage of number of myonuclei per fiber. *P < 0.05, **P < 0.01

Pax7/Ki67 double staining of cryosections from rat IgG and MR16-1-treated dKO mice showed similar levels of proliferated satellite cells (Pax7/Ki67 double-positive), which were greater compared with those of wild-type mice (1.02 ± 0.15 in rat IgG vs. 1.06 ± 0.18 cells/400× field in MR16-1-treated, and 0.07 ± 0.06 cells/400× field in wild-type, Fig. 4a, b). MR16-1 treatment significantly increased the number of quiescent satellite cells (Pax7+/Ki67−) per field (5.56 ± 0.39 in rat IgG vs. 6.54 ± 0.82 cells/400× field in MR16-1-treated, *P* < 0.05 and 3.07 ± 0.32 cells/400× field in wild-type, Fig. 4a, b).

The percentage area of regenerating muscle fibers, which expresses eMyHC was significantly increased upon MR16-1 treatment (2.4% ± 0.7%) relative to the rat IgG-treated group (1.5% ± 0.4%, *P* < 0.05, Fig. 4c, d). While the numbers of eMyHC-positive regenerating fibers were similar (Fig. 4e), the average fiber size was significantly larger in MR16-1-treated dKO mice (*P* < 0.05, Fig. 4f). These results were in agreement with a significant increase in mRNA levels of *myh3* in the MR16-1-treated group (*P* < 0.01, Fig. 4g). We also assessed the contribution of satellite cells to fiber diameters by counting myonuclei per fiber (Fig. 4h). Myonuclei were identified by applying anti-laminin α2 antibody and DAPI staining, and about 300 to 500 fibers per section were counted. MR16-1-treated dKO mice had significantly less muscle fibers, contained one nucleus (*P* < 0.05), and had a possible trend in more muscle fibers containing 4 and 5 nuclei (*P* = 0.08 and *P* = 0.14, respectively, Fig. 4h).

### Modulated inflammatory response and reduced fibrosis by MR16-1

**Fig. 5:**
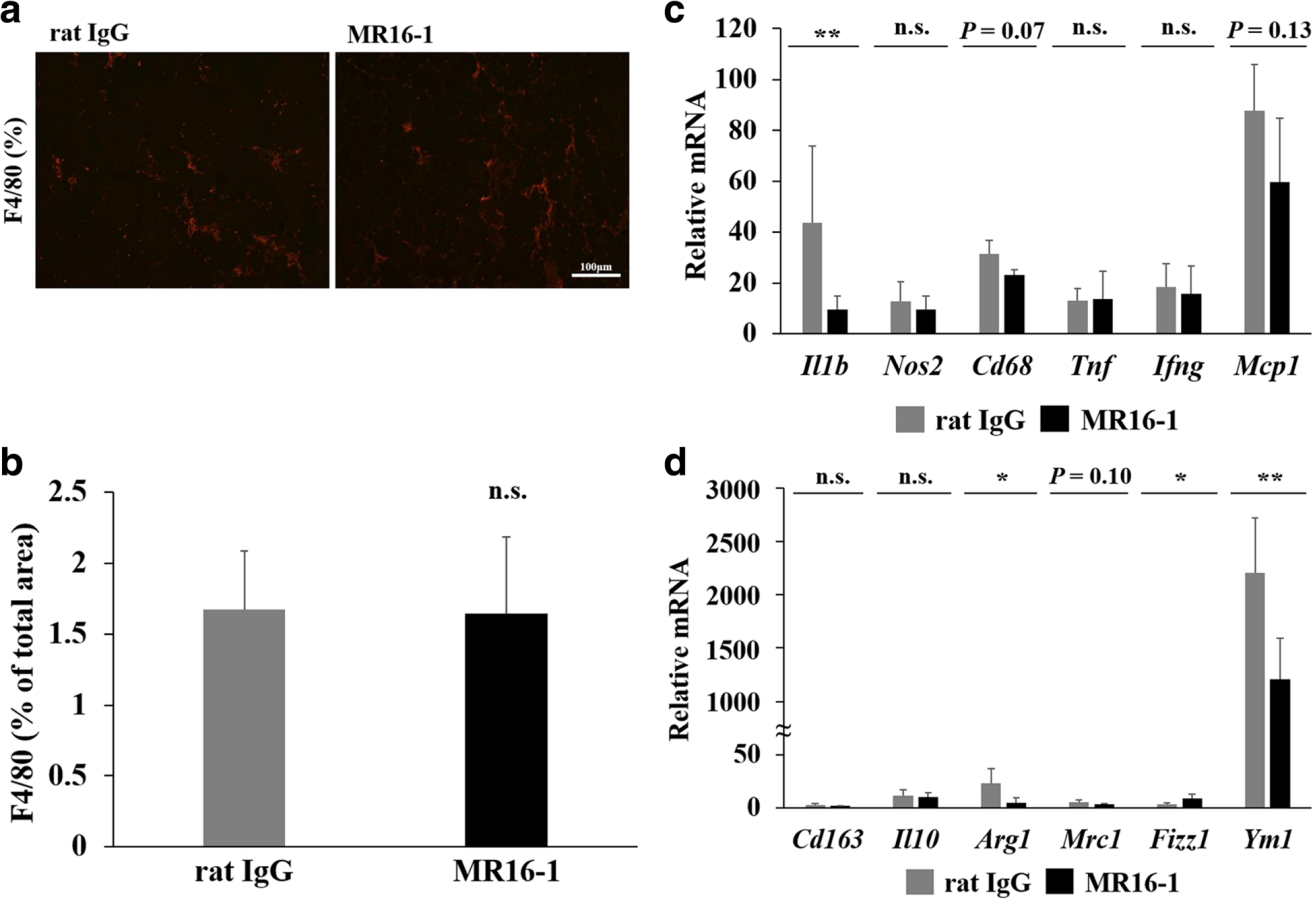
Histological and real-time PCR analyses of inflammatory response in the quadriceps muscle. **a** F4/80-positive inflammatory area of the quadriceps muscle from dKO mice. **b** The percentage of F4/80-positive area per total muscle fiber area was quantified. **c** Gene expression levels of pro-inflammatory cytokines (*Il1b*, *Nos2*, *Cd68*, *Tnf*, *Ifng*, and *Mcp1*, normalized by *Lbr*) were measured. **d** Gene expression levels of anti-inflammatory cytokines (*Cd163*, *Il10*, *Arg1*, *Mrc1*, *Fizz1*, and *Ym1*, normalized by *Lbr*) were measured. The levels were expressed as fold increase of wild-type mice (*n* = 3–4 per group). *P < 0.05, **P < 0.01

Inflammation was analyzed by the immunostaining of the F4/80-positive area per total cryosectional area of the quadriceps muscle. F4/80 is a standard marker of mature macrophages, and we found approximately 1.6% of the total area were F4/80-positive macrophages. There was no difference in total area of inflammation in rat IgG and MR16-1-treated mice (Fig. 5a, b). We further assessed the expression of pro-inflammatory (M1) and anti-inflammatory (M2) cytokines by real-time PCR. All the data were shown as the fold increase versus the values of wild-type mice. Pro- and anti-inflammatory genes were dramatically upregulated in dKO mice compared with wild-type mice (Fig. 5c, d). Among mRNA levels of M1 macrophage markers, *Il1b* was significantly decreased (*P* < 0.01), and *Cd68* and *Mcp1* were reduced with a possible trend (*P* = 0.07 and *P* = 0.13, respectively, Fig. 5c) by MR16-1 treatment. IL-6R blockade also reduced *Arg1* (*P* < 0.05), *Ym1* (*P* < 0.01), and *Mrc1* (*P* = 0.10) and significantly increased *Fizz1* (*P* < 0.05), the major M2 cytokines (Fig. 5d).

**Fig. 6:**
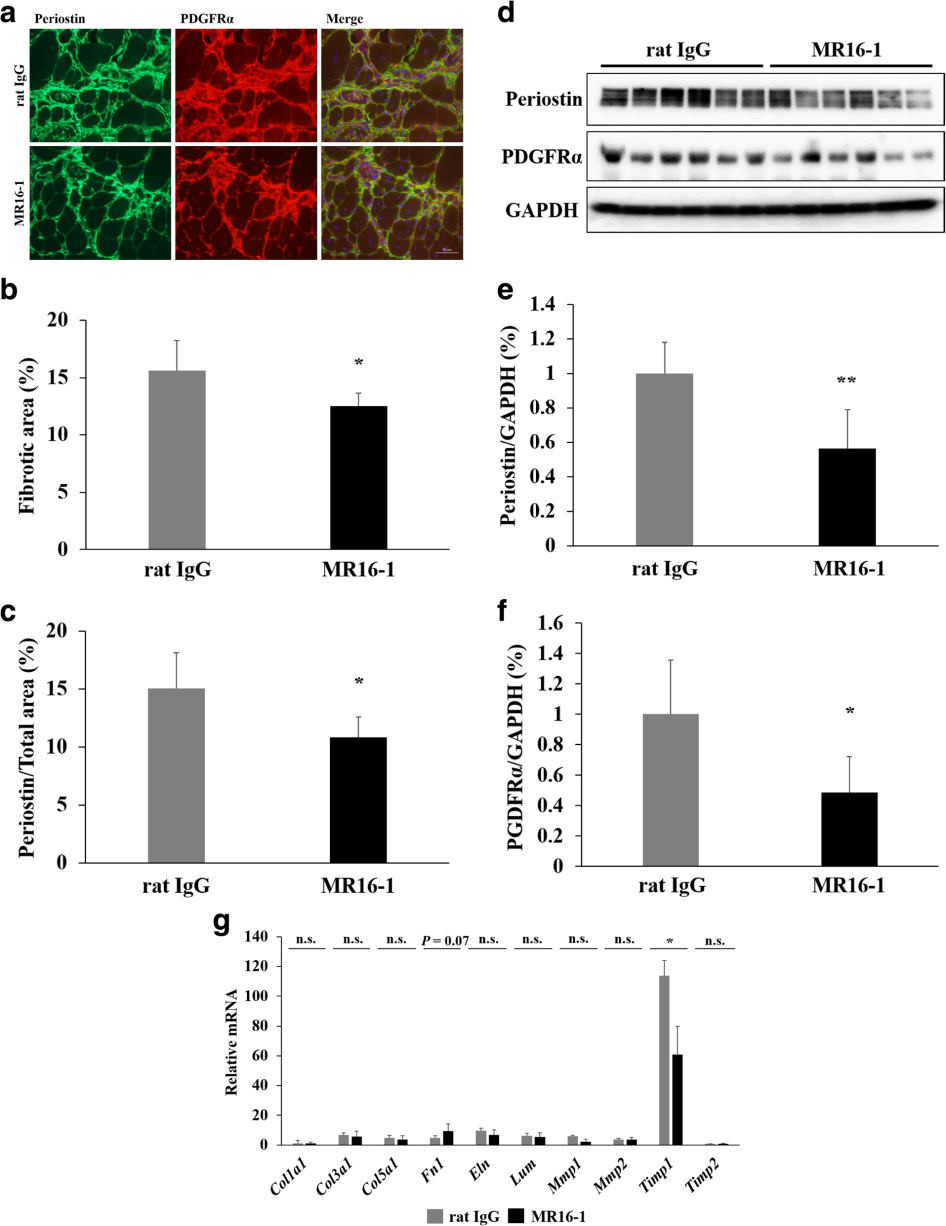
The effects of MR16-1 treatment on fibrosis in the quadriceps muscle. **a** A representative image of double staining of periostin and PDGFRα in the quadriceps muscle. PDGFRα-positive cells were co-localized with periostin-positive fibrotic area. **b** The quantification of fibrotic area of muscle sections positive for Masson’s trichrome, and **c** periostin. **d** Western blot analysis of periostin and PDGFRα expression (*n* = 6 per group). **e** The expression levels of periostin and **f** PDGFRα, normalized by GAPDH, of dKO mice treated with rat IgG or MR16-1 were compared. **g** The levels of fibrosis-related genes (*Col1a1*, *Col3a1*, *Col5a1*, *Fn1*, *Eln*, *Lum*, *Mmp1*, *Mmp2*, *Timp1*, and *Timp2*, normalized by *Lbr*) were measured. The levels were expressed as fold increase of wild-type mice (*n* = 3–4 per group). *P < 0.05, **P < 0.01

As periostin is highly accumulated in fibrotic areas of the extracellular matrix (ECM) of the dKO skeletal muscle, we quantified fibrosis using an anti-periostin antibody. Moreover, fibro/adipogenic progenitor cells, expressing the platelet-derived growth factor receptor-α (PDGFRα), contribute to the development of fibrosis and disease progression in an advanced stage of dystrophic muscle. We performed double immunostaining of periostin and PDGFRα and found that these proteins were similarly accumulated in fibrotic areas in the quadriceps muscle of dKO mice (Fig. 6a). We quantified the fibrotic area in skeletal muscle sections stained with Masson’s trichrome (Fig. 3a) and periostin (Fig. 6a) and showed that the fibrotic area was significantly decreased by MR16-1 treatment (*P* < 0.05, Fig. 6b, c). In addition, the quantitative analyses of the expression of periostin and PDGFRα by Western blot revealed that both proteins were significantly decreased by MR16-1 treatment (*P* < 0.01 and *P* < 0.05, respectively, Fig. 6d–f). To investigate the roles of IL-6R blockade on inhibition of skeletal muscle fibrosis, we analyzed the expression of fibrosis-related genes. The genes related to ECM were not changed by MR16-1 treatment except a trend increase in the expression of *Fn1* (*P* = 0.07, Fig. 6g). Matrix metalloproteinases (MMPs), involved in the turnover of ECM components, and natural inhibitors of metalloproteinases (TIMPs) are also important contributors of fibrosis. We found that the gene expression of *Timp1*, which was approximately 110 times increased in rat IgG dKO mice compared with wild-type mice, was significantly decreased in the quadriceps muscle of dKO mice by IL-6 blockade (*P* < 0.05, Fig. 6g).

### Effects of MR16-1 on pathology and IL-6 signaling pathway in cardiorespiratory muscles

**Fig. 7:**
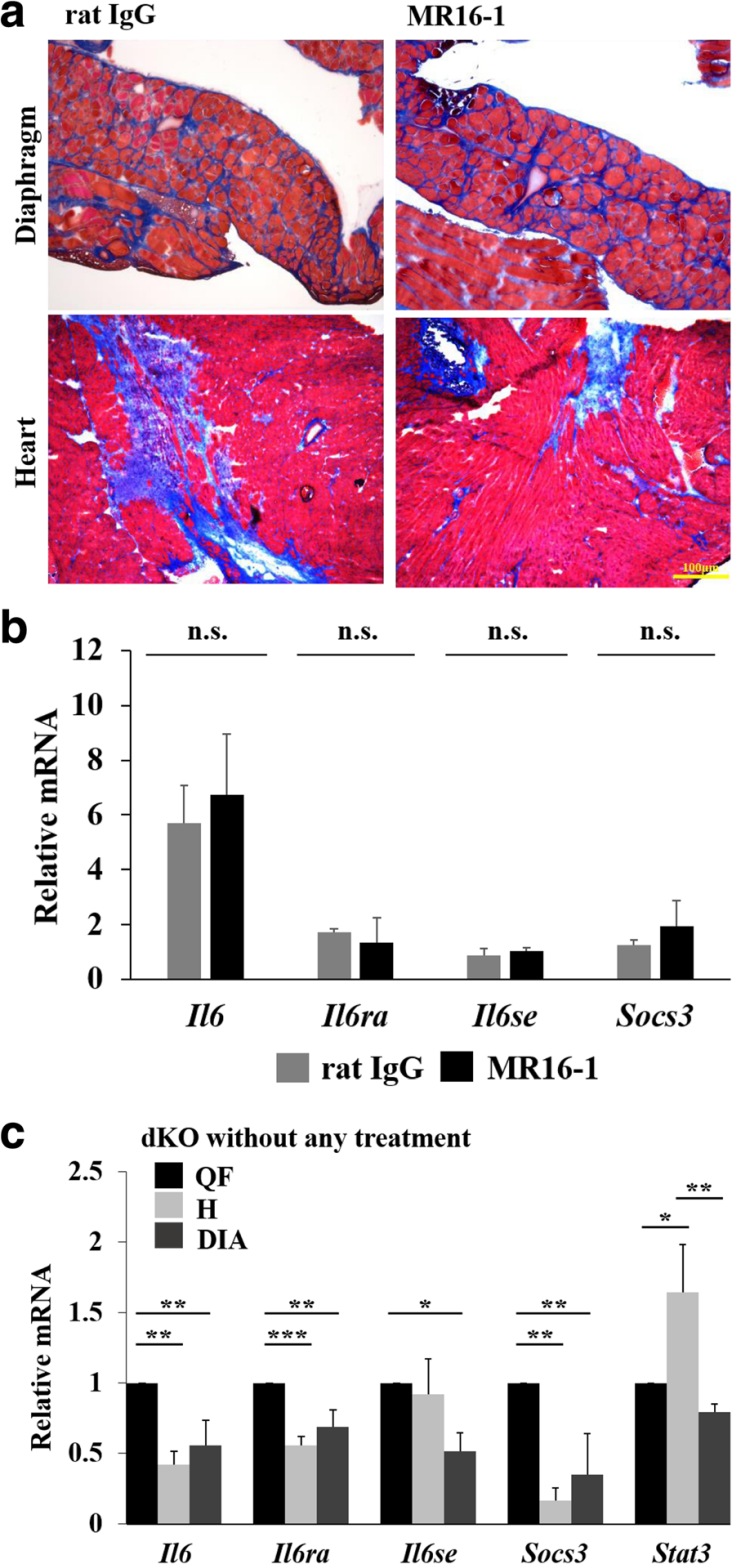
IL-6 signaling pathway in the diaphragm and cardiac muscle. **a** Representative images of H&E and Masson’s trichrome staining of the diaphragm and heart of dKO mice treated with rat IgG or MR16-1. **b** Gene expression levels of *Il-6*, *Il-6ra*, *Il-6se*, and *Socs3* in cardiac muscle were measured by real-time PCR. All samples were normalized using the gene expression levels of *Tbp*, and expressed as fold increase of wild-type mice (*n* = 3–4 per group). **c** The mRNA levels of genes associated with IL-6 (*Il-6*, *Il-6ra*, *Il-6se*, *Socs3*, and *Stat3*) were compared among the quadriceps muscle, diaphragm, and cardiac muscle of the same normal (non-treated) dKO mice at 90 days of age (*n* = 3). *P < 0.05, **P < 0.01, ***P < 0.001

Adult dKO mice have exacerbated muscle pathology not only in the skeletal muscle but also in cardiac and respiratory muscles. Masson’s trichrome staining as well as immunostaining by an anti-periostin antibody (data not shown) showed no improvement in the fibrotic area in the cardiorespiratory muscles of MR16-1-treated dKO mice (Fig. 7a). Even though the levels of *Il6* gene was upregulated in dKO mice compared with wild-type mice, the expression of IL-6-related genes was not changed in the cardiac muscle upon IL-6R blockade (Fig. 7b). We hypothesized that the accumulation of fibrosis in cardiorespiratory muscles might be triggered by a different mechanism. To support this speculation, we compared the IL-6 signaling pathway in the quadriceps, heart, and diaphragm from the same non-treated dKO mice at 90 days of age. Gene expression levels of *Il6*, *Il6ra*, and *Socs3* in the quadriceps muscle were significantly higher than the heart and diaphragm (Fig. 7c). The expression levels of those genes were similar in the heart and diaphragm. *Stat3* gene expression was significantly higher in the cardiac muscle than the quadriceps and diaphragm. Phosphorylated STAT3 and total STAT3 levels in the skeletal and cardiac muscles from dKO mice treated with rat IgG and MR16-1 were compared with samples from wild-type mice (Additional file 1: Figure S2). There was no detectable STAT3 activation in the cardiac muscle of all the mice tested by Western blot analysis, in contrast to the skeletal muscle from rat IgG-treated dKO mice.

## Discussion

In addition to recent advancements in research into gene therapies for DMD patients [23], anti-inflammatory drugs are expected to have beneficial effects for delaying disease progression [24]. IL-6 is present at high levels in the blood and skeletal muscle of DMD patients and animal models, and chronic upregulation of IL-6 plays a crucial role in the pathogenesis of DMD, such as causing severe muscle degeneration, inflammation, and accumulation of non-functional fat and fibrotic tissues. Exacerbation of the dystrophic phenotype by increased circulating IL-6 levels in mdx/IL-6 transgenic mice also supports that IL-6 accelerates disease progression [13]. The anti-IL-6R antibody has been successfully used for the treatment of rheumatoid arthritis and inflammatory diseases, and may be applicable for the treatment of other immune-mediated diseases including cancer [25, 26]. We hypothesized that IL-6R blockade may be an effective therapeutic option for DMD.

We examined the effects of the MR16-1 antibody on chronic stages of the dystrophic phenotype in dKO mice. Previous studies focused on the effectiveness of MR16-1 treatment in reducing inflammation in the skeletal muscle and improving the muscle function of mdx mice, but contradicting results have been reported, partly due to the analyses of different muscles, such as the gastrocnemius muscle [14] or diaphragm [15], at different ages. The amount of MR16-1 antibody injection was also different; however, the most significant limitation of these studies was that they analyzed the efficacy of the antibody using mdx mice. Mdx mice have high circulating and skeletal muscle IL-6 levels, but their clinical phenotype is relatively mild with high muscle regenerating capacity, as well as a nearly normal lifespan. The upregulation of utrophin plays a pivotal role in the milder phenotypes of mdx mice, and the loss of both dystrophin and utrophin in dKO mice causes more severe and progressive phenotypes. In this study, we clearly demonstrated the therapeutic efficacy of IL-6R blockade on the skeletal muscle, particularly on promoting muscle regeneration, increasing muscle fiber diameter, and reducing fibrosis, but not the efficacy on cardiorespiratory muscles from dKO mice.

Based on previous studies investigating MR16-1 as a treatment for murine models of inflammatory diseases, we expected that the therapeutic effect of MR16-1 administration would be maximized when treatment started as early as possible. The dose of MR16-1 administered to dKO mice was determined from the results of a high-dose tolerance experiment using BWF1 (NFZ/WF1) mice (a model of autoimmune disease) [27]. All rat IgG and MR16-1-treated dKO mice were alive until 90 days of age, body weights were similar to non-treated dKO mice, and no abnormal morphologies were observed in the internal organs. A previous study also showed that there were no adverse effects of MR16-1 administration on sexual maturation or development of the immune and skeletal systems of juvenile wild-type [28].

IL-6R blockade increased circulating IL-6 protein levels, but the IL-6 signaling pathway was successfully inhibited in the skeletal muscle of dKO mice, as shown by significant decreased gene expression levels of *Il6* and *Socs*3 and reduction in phosphorylated STAT3 protein levels. Increased serum IL-6 levels upon MR16-1 treatment have also been reported in other studies using murine models of various diseases, although the anti-IL-6R antibody is effective in ameliorating their phenotypes [29, 30]. Furthermore, a single injection of tocilizumab causes an increase in circulating IL-6 levels but does not affect IL-6 gene expression in the liver or other organs from monkeys with experimental arthritis [31]. Clinical data also demonstrate the improvement of rheumatoid arthritis by IL-6R blockade despite increased blood IL-6 levels after the administration of MR16-1 [32]. The precise mechanism has not been clarified; however, a study conducted by Osaka University and Chugai Pharmaceutical Company explains that IL-6 signaling is successfully inhibited by the anti-IL-6R antibody, and there is a tendency of increase in serum IL-6 levels because free IL-6 cannot make a complex with IL-6R, and clearance of the cytokine by the receptor is also restricted [33].

Significant reduction in serum CK levels and the number of necrotic fibers indicated the amelioration of muscle fiber degeneration in dKO mice treated with MR16-1. No differences were observed in total body weight at 90 days of age between rat IgG and MR16-1-treated groups. Complete loss of both dystrophin and utrophin in mice also leads to the progression of spinal deformity, severe bone loss, and impaired bone remodeling [21]. Some MR16-1-treated mice demonstrated an improvement in KI; however, we did not determine the effects of the anti-IL-6R antibody on the bone quality of dKO mice.

The major therapeutic evidence of MR16-1 treatment was the improvement in the percentage area of regenerating muscle fibers and the size of mature fibers. The anti-IL-6R antibody successfully inhibited the IL-6 signaling pathway in the skeletal muscle, particularly on STAT3 activation, which is a downstream effector of IL-6. Activation of STAT3 is reported to be a negative regulator of satellite cell activation in muscle repair [34, 35]. In addition, the inhibition of intramuscular STAT3 improves muscle regeneration and enlarges muscle fiber diameter in mdx mice [35]. On the other hand, genetic deletion of STAT3 (satellite cells-specific *Stat3* KO mice) negatively regulates proliferation and self-renewal of satellite cells after CTX-induced injury [36]. These controversial results are due to the different approaches to inhibit STAT3 in the skeletal muscle, transient inhibition by pharmacological inhibitors or siRNA, or genetic deletion. As Zhu H et al. [36] suggests, a direct and long-term treatment of STAT3 inhibition might have adverse effects on muscle satellite cells of DMD patients. Our results demonstrated that the anti-IL-6R antibody successfully inhibited phosphorylated STAT3 levels in the dKO skeletal muscle; however, total STAT3 levels were not affected. The inhibitory effect of MR16-1 on the phosphorylation of STAT3 was clearly demonstrated in a previous study using a cardiotoxin (CTX)-induced muscle injury model [37]. Activated STAT3 was significantly upregulated in the wild-type skeletal muscle at 3 days after CTX-induced injury, and MR16-1 administration accelerated muscle regeneration. We confirmed that gene expression levels of *myh3* and the eMyHC-positive area in the dKO skeletal muscle were significantly increased by MR16-1 injection. The improvement in muscle regeneration of MR16-1-treated mice resulted in an increase in the diameter of larger muscle fibers. Pax7-positive satellite cells from the dKO skeletal muscle dramatically reduce the ability of proliferation compared with mdx mice. Furthermore, a decrease in the number of satellite cells was observed, which would result in impaired muscle regeneration and increased inflammation in dKO mice in an age-dependent manner [38, 39]. In this study, we demonstrated that quiescent satellite cells (Pax7+/Ki67−) were significantly preserved while the number of activated satellite cells (Pax7+/Ki67−) did not differ in dKO mice by MR16-1 treatment. The larger number of myonuclei per fiber by MR16-1 treatment supported the active satellite cell proliferation during the regeneration process, which resulted in increased muscle fiber diameter.

We hypothesized that the anti-IL-6R antibody is beneficial for reducing skeletal muscle inflammation in dKO mice; however, contrary to our hypothesis, we did not observe any differences in the F4/80-positive area upon MR16-1 treatment. Compared with mdx mice, there was a substantial amount of fibrotic tissue in the skeletal muscle of dKO mice. When compared, at the same age, the quadriceps muscle of mdx mice has a higher percentage of F4/80-positive inflammatory area than that of control dKO mice [40]. The action of pro- and anti-inflammatory cytokines in dystrophic muscle is distinguished in different ages and phases of the disease [41, 42], so we analyzed the mRNA levels of inflammatory cytokines. Our data indicated that most of the pro- and anti-inflammation-related genes were upregulated in the dKO skeletal muscle compared with wild-type mice; therefore, decrease in both pro- and anti-inflammatory cytokines by IL-6R blockade represented the reduction of overall activity of F4/80-positive macrophage. We interpreted that the increase in quiescent satellite cell (Pax7+/Ki67−) number in MR16-1-treated dKO mice is potentially achieved by reducing the activity of inflammatory macrophages. Future studies should address the direct effects of inhibition of activated STAT3 in inflammatory cells on satellite cells from dystrophic muscles.

An advantage of examining the effectiveness of MR16-1 using dKO mice is the presence of fibrosis in their skeletal muscle. The skeletal muscle of patients in advanced stages of DMD also contains fat and fibrotic tissue, with few remaining skeletal muscle fibers [4, 43]. Our data demonstrated that skeletal muscle fibrosis in dKO mice was significantly inhibited by MR16-1 treatment, as shown by a reduced positive area for Masson’s trichrome staining and periostin accumulation in ECM. Moreover, PDGFRα-positive fibro/adipogenic progenitor cells contribute to the development of fibrosis in the advance stage of DMD [44–46]. We confirmed that PDGFRα-positive cells were co-localized with the periostin-positive fibrotic area in dKO mice, and the Western blot analysis showed the significant decrease in PDGFRα in the quadriceps muscle of dKO mice by MR16-1 treatment. Most of the fibrosis-related genes were upregulated, especially *Timp1*, in dKO mice, and MR16-1 treatment significantly decreased the levels of *Timp1*. The upregulation of the gene is also found in the skeletal muscle of DMD patients, and an unbalance in matrix metallopeptidase 1 (MMP1)/TIMP metallopeptidase inhibitor 1 (TIMP-1) ratio is a distinguished feature in DMD muscle from dermatomyositis or polymyositis which are rarely developed fibrosis in the skeletal muscle [47]. A previous study demonstrates the IL-6 signaling pathway induces TIMP-1 production in synovial fibroblasts [48]. The regulation of TIMP-1 by IL-6 in skeletal muscle fibrosis should be further studied whether IL-6R blockade could directly inhibit the development of fibrosis in DMD muscle.

Cardiac dysfunction is a frequent complication of DMD and a common cause of death. Regarding the skeletal muscle, dKO mice have a severer phenotype in cardiorespiratory muscles than mdx mice, particularly in the development of fibrosis [20]. Despite the beneficial effects of IL-6R blockade on the dKO skeletal muscle, we did not observe any significant therapeutic effects in the diaphragm and cardiac muscles. The fibrotic area in the cardiac muscle and diaphragm were not improved, and the area of regenerating muscle fibers in the diaphragm was similar. Surprisingly, the IL-6 signaling pathway in the cardiac muscle was not affected by anti-IL-6R antibody treatment even though the gene expression of *Il6* was upregulated compared with cardiac muscle from wild-type mice. We compared the IL-6 signaling pathway in the quadriceps, heart, and diaphragm from the same non-treated dKO mice at 90 days of age, and observed an enhanced IL-6 pathway in the skeletal muscle. Concomitantly, phosphorylated STAT3 levels were not detected even in rat IgG dKO cardiac muscle. Taken together, the skeletal muscle and cardiac muscle in dKO mice appear to have a different pathogenic mechanism, particularly regarding the IL-6 signaling pathway.

There are still limitations in this study that the effects of MR16-1 treatment on muscle function and the survival curve of dKO mice were not investigated. Importantly, these parameters are necessary to determine before conducting a clinical trial. Unlike mdx mice, dKO mice are vulnerable and have a short lifespan; however, they do not genetically mirror human DMD. Thus, mdx/utrophin-heterozygous mice may be a more useful DMD model for a further study to evaluate a longer-term treatment of IL-6R blockade, particularly on analyzing muscle function, a time course change in inflammatory response, and the survival curve [49, 50].

## Conclusions

We clearly demonstrated that treatment with MR16-1 antibody successfully inhibited the activated IL-6 signaling pathway in the skeletal muscle. Blocking this pathway resulted in decreased muscle damage, improved muscle fiber regeneration, enlarged fiber diameter, and reduced fibrosis in dKO mice at an advanced stage. Although IL-6R blockade did not exert any therapeutic effects on cardiorespiratory muscles, administration was safe and beneficial for delaying the disease progression of skeletal muscle pathology. As the anti-IL-6R antibody is already an approved treatment for children with systemic juvenile idiopathic arthritis, it is potentially applicable as a treatment for children with DMD.

## Additional file

Additional file 1: Table S1. Primer sequences used for quantification of gene expression. **Figure S1.** A representative image of measuring axes of skeletal muscle fibers using IN Cell Analyzer 2200. A muscle fiber is first fit to a bounding ellipse. The shorter axis is automatically recognized as the minor axis and the longer is the major axis. The minor axis was used to analyze a distribution of the quadriceps muscle fiber diameter. **Figure S2.** Western blot analysis. The protein expression of pSTAT3, STAT3, periostin, PDGFRα and GAPDH in the quadriceps muscle (QF) and heart (H) of wild-type mice, and dKO mice treated with rat IgG or MR16-1. (DOCX 1617 kb)

## Abbreviations

CK: Creatine kinase
CTX: Cardiotoxin
dKO: Dystrophin/utrophin double-knockout
DMD: Duchenne muscular dystrophy
eMyHC: Embryonic myosin heavy chain
H&E: Hematoxylin and eosin
i.p.: Intraperitoneally
IL-6: Interleukin-6
IL-6R: Interleukin-6 receptor
JAK-STAT: Janus kinase/signal transducer and activator of transcription
KI: Kyphotic index
KO: Knockout
MCIs: Myofibers containing internal nuclei
MMPs: Matrix metalloproteinases
PDGFRα: The platelet-derived growth factor receptor-α
TIMPs: Inhibitors of metalloproteinases
