# Supplementary figures and images for: Treatment with the anti-IL-6 receptor antibody attenuates muscular dystrophy via promoting skeletal muscle regeneration in dystrophin-/utrophin-deficient mice
Source: Skelet Muscle. 2017 Oct 27;7:23. doi: 10.1186/s13395-017-0140-z (PMC5660454; doi:10.1186/s13395-017-0140-z)

Table S1


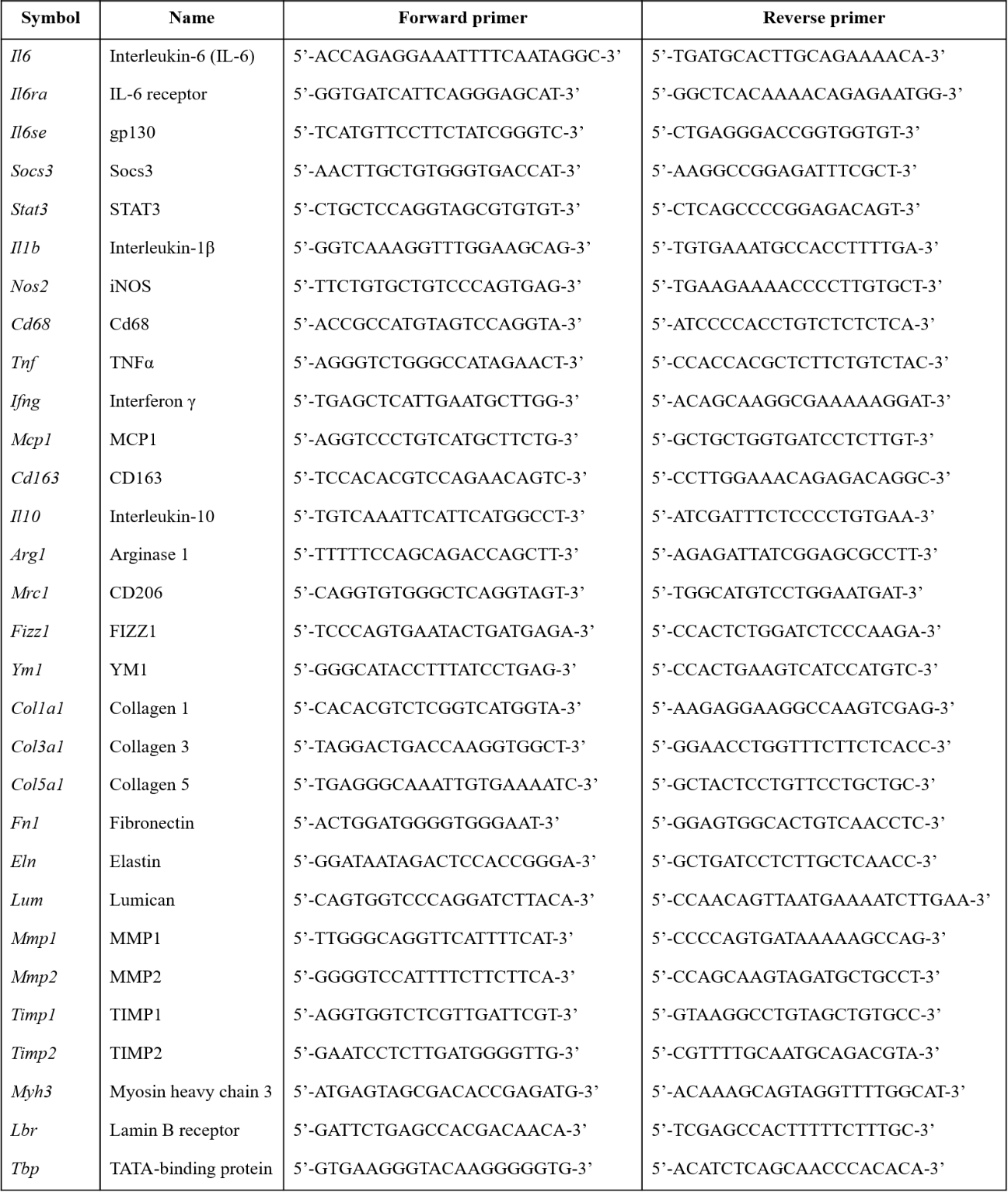


Figure S1


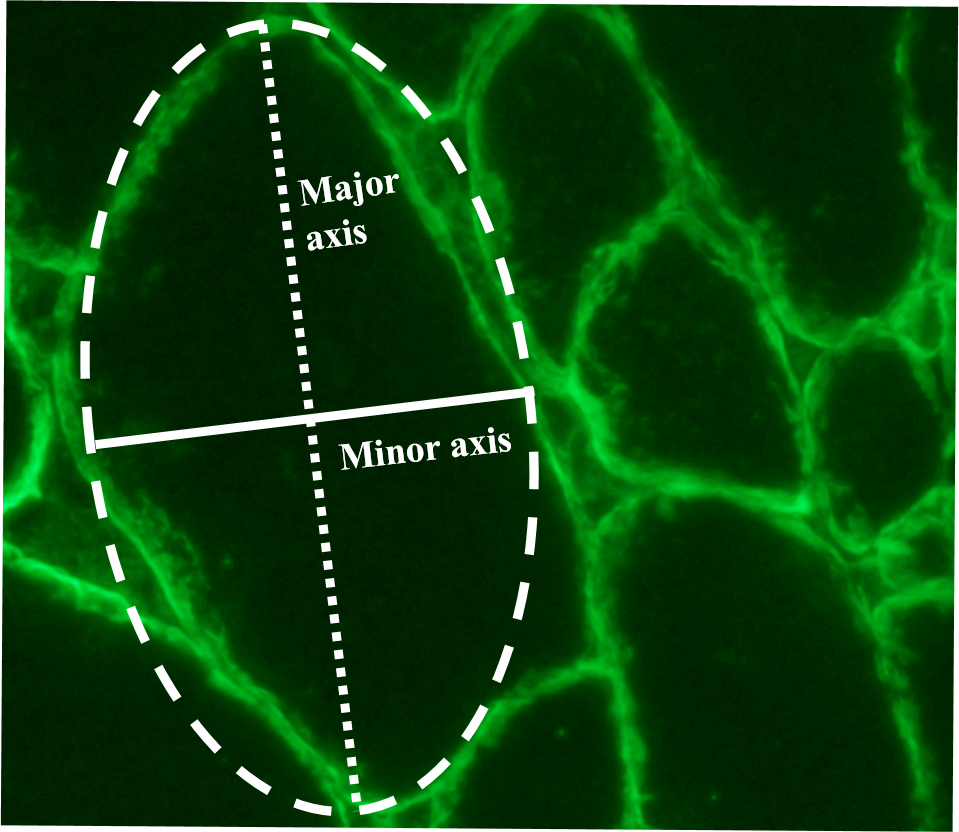


Figure S2


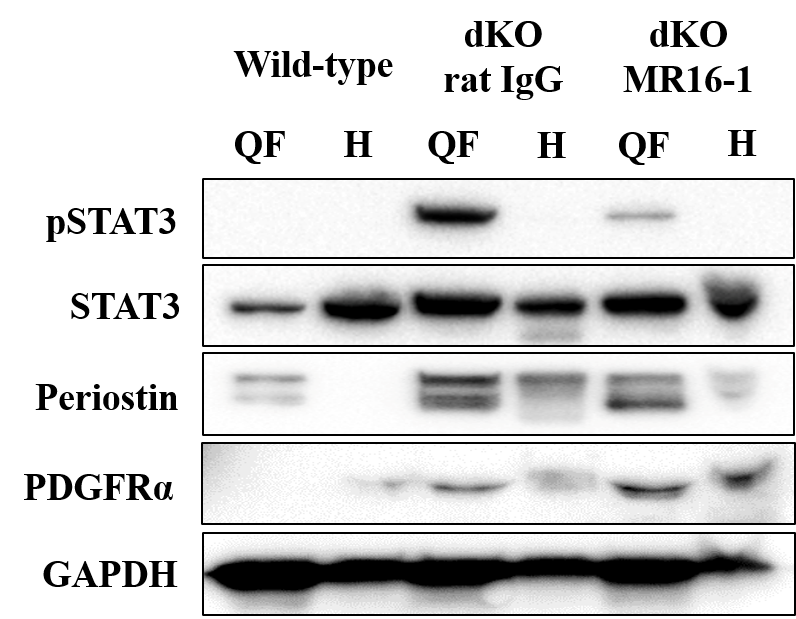

Supplement: Additional file 1: Table S1. — Primer sequences used for quantification of gene expression. Figure S1. A representative image of measuring axes of skeletal muscle fibers using IN Cell Analyzer 2200. A muscle fiber is first fit to a bounding ellipse. The shorter axis is automatically recognized as the minor axis and the longer is the major axis. The minor axis was used to analyze a distribution of the quadriceps muscle fiber diameter. Figure S2. Western blot analysis. The protein expression of pSTAT3, STAT3, periostin, PDGFRα and GAPDH in the quadriceps muscle (QF) and heart (H) of wild-type mice, and dKO mice treated with rat IgG or MR16-1. (DOCX 1617 kb) [file 13395_2017_140_MOESM1_ESM.docx]
